# Effectiveness of clinical criteria in directing patient flow from the emergency department to a medical assessment unit in Queensland, Australia: a retrospective chart review of hospital administrative data

**DOI:** 10.1186/s12913-021-06537-7

**Published:** 2021-05-29

**Authors:** Sonya Osborne, Helen Cleak, Nicole White, Xing Lee, Anthony Deacon, Julian W M de Looze

**Affiliations:** 1 School of Nursing and Midwifery, Centre for Health Research, Institute of Resilient Regions, University of Southern Queensland, 4305 Ipswich, Queensland Australia; 2 Australian Centre for Health Services Innovation, School of Public Health and Social Work, Queensland University of Technology, Queensland 4059 Kelvin Grove, Australia; 3 Department of Community and Clinical Health, La Trobe University, 3086 Melbourne, Victoria Australia; 4 Department of Internal Medicine and Aged Care, Metro North Hospital and Health Service, Royal Brisbane and Women’s Hospital, 4029 Herston, Queensland Australia; 5 School of Medicine, The University of Queensland, 4067 St. Lucia, Queensland Australia; 6 School of Electrical Engineering and Computer Science, Queensland University of Technology, Queensland 4059 Kelvin Grove, Australia

**Keywords:** Emergency presentations, Medical admissions, Medical assessment units, MAU, Short-stay unit

## Abstract

**Background:**

Medical Assessment Units (MAUs) have become a popular model of acute medical care to improve patient flow through timely clinical assessment and patient management. The purpose of this study was to determine the effectiveness of a consensus-derived set of clinical criteria for patient streaming from the Emergency Department (ED) to a 15-bed MAU within the highly capacity-constrained environment of a large quaternary hospital in Queensland, Australia.

**Methods:**

Clinically coded data routinely submitted for inter-hospital benchmarking purposes was used to identify the cohort of medical admission patients presenting to the ED in February 2016 (summer) and June 2016 (winter). A retrospective review of patient medical records for this cohort was then conducted to extract MAU admission data, de-identified patient demographic data, and clinical criteria. The primary outcome was the proportion of admissions that adhered to the MAU admission criteria.

**Results:**

Of the total of 540 included patients, 386 (71 %) patients were deemed to meet the MAU eligibility admission criteria. Among patients with MAU indications, 66 % were correctly transferred (95 % CI: 61 to 71) to the MAU; this estimated sensitivity was statistically significant when compared with random allocation (*p*-value < 0.001). Transfer outcomes for patients with contraindications were subject to higher uncertainty, with a high proportion of these patients incorrectly transferred to the MAU (73 % transferred; 95 % CI: 50 to 89 %; *p*-value = 0.052).

**Conclusions:**

Based on clinical criteria, approximately two-thirds of patients were appropriately transferred to the MAU; however, a larger proportion of patients were inappropriately transferred to the MAU. While clinical criteria and judgement are generally established as the process in making decisions to transfer patients to a limited-capacity MAU, our findings suggest that other contextual factors such as bed availability, time of day, and staffing mix, including discipline profile of decision-making staff during ordinary hours and after hours, may influence decisions in directing patient flow. Further research is needed to better understand the interplay of other determinants of clinician decision making behaviour to inform strategies for improving more efficient use of MAUs, and the impact this has on clinical outcomes, length of stay, and patient flow measures in MAUs.

## Background

The demand on hospital services has been consistently rising over the past decades worldwide, with a disproportionate increase in emergency department (ED) presentations leading to overcrowding, treatment delays, and adverse patient outcomes [1–3]. Increasingly, research studies have demonstrated the association of seasonal variations and higher presentations to hospital, with peaks in winter, particularly from cardiovascular and respiratory disease [4–6].

Decision-making in acute care, particularly within the ED context, occurs in a pressurized, time-critical environment and is often exacerbated by target driven pressures and under resourced staffing. This, coupled with the increasing numbers of patients accessing the health system through the ED, can lead to patients waiting for assessment, lack of timely communication, delays in treatment, and overcrowding – which in turn, can increase risk of complications and medical error and contribute to poor patient outcomes [7, 8]. One solution to this situation has been the establishment of Medical Assessment Units (MAUs) designed to streamline the admission process and expedite rapid and comprehensive multidisciplinary assessment of acute medical patients while enhancing capacity of EDs to off-load non-critically ill medical patients. The emergence of MAUs as an alternative to standard hospital admission is one approach designed to improve systems of care and patient flow from the ED through rapid assessment and early decision making by senior ED clinicians [9].

MAUs are typically designed to optimise flow and clinical outcomes for patients requiring urgent or emergent medical hospital admission [10, 11]. Such models of care include streaming of patients to appropriate clinical locations based on complexity and likely disposition. The design of MAUs varies considerably with respect to admission and discharge policies, as well as the model of care provided to patients during their MAU stay. MAUs can be physically co-located in or near the emergency department or located elsewhere in the hospital and typically have increased staffing numbers and skill mix to cater for more rapid patient assessment, discharge planning and management of acute illness [10, 11].

MAUs have been frequently established to not only improve health service performance and patient outcomes but to assist with the achievement of hospital emergency access targets. Published evaluations of the MAU model report that MAUs appear to reduce the need for admission to hospital care, reduce hospital length of stay (LOS), free-up ED beds for patients that require more extensive workup, and minimise the effects of overcrowding and treatment delays in the ED [1, 10, 12–15]. Consequently, appropriate patient streaming to MAUs can improve a hospital’s performance on key health care indicators without compromising the quality of patient care [11].

Decisions to stream to MAUs are generally based on a set of essential principles, such as a higher ratio of senior medical staff, established strict clinical assessment and treatment protocols, prioritised investigations and urgent treatment coordinated in one clinical area. Adherence to these principles leads to patients benefiting from more timely and appropriate clinical care [7, 11, 13, 14]. While the tangible benefits of MAUs are laudable, the relative importance of other factors that may impact on the effective utilization and organization of the service remains relatively unexplored.

A two-phased research study was undertaken to determine the effectiveness of clinical criteria for patient streaming from the ED to a MAU within the complex environment and patient flow challenges of a highly capacity-constrained large quaternary hospital and to identify and explore the influence of potential explanatory factors for deviations in patient streaming disposition. This paper presents the findings from the first phase, a retrospective medical record review of a hospital administrative data set to describe variables associated with medical patient transfers from the ED. The *REporting of studies Conducted using Observational Routinely-collected health Data (RECORD)* will be used to report the findings of the study [16].

## Methods

### Aim

The aim of this study was to determine the effectiveness of a consensus-derived set of clinical criteria for patient streaming from the ED to a MAU within a highly capacity-constrained environment of a large quaternary hospital.

### Research Design

 A retrospective medical record review of hospital administrative data was used to address the research aim. The study was assessed by The Prince Charles Hospital Human Research Ethics Committee (approval number: HREC/16/QPCH/505) and deemed a quality improvement initiative and did not require ethical approval.

### Setting

The study was conducted at a large, 929-bed, quaternary referral hospital in Queensland Australia. In 2015, the hospital admitted 100,149 patients, with 74,399 emergency department presentations [17]. The 15-bed MAU is designed to improve access for emergently admitted patients entering the health system through the ED. Using the revised peer grouping data for Australian public and private hospitals [18], the study hospital was typical of acute public hospitals considered principal referral hospitals with MAUs in Australia as presented in Table 1.

**Table 1:** Comparison of study hospitals with Australian and Queensland principal referral hospitals on four key hospital indicators (2016–2017)

	Australia	Study Hospital^a^
Hospital Indicators	Principal referral hospitals^b^ (*n* = 29)	Principal referral hospital
Average acute weighted separations (range)^c^	74,631 (43,006-108986)	77,543
Emergency department presentations (range)	61,072 (34,645 − 117,737)	76,533
Average hospital length of stay in days (range)^d^	3.5 (2.5–4.7)	2.9

^a^Source: internal study site data

^b^Principal referral hospitals provide a very broad range of services, including some very sophisticated services, and have very large patient volumes. Most include an intensive care unit, a cardiac surgery unit, a neurosurgery unit, an infectious diseases unit and a 24-hour emergency department.

^c^Separations are an episode of care for an admitted patient, which can be a total hospital stay (from admission to discharge, transfer or death) or a portion of a hospital stay beginning or ending in a change of type of care (for example, from acute care to rehabilitation); it reflects admitted patient activity.

^d^The length of stay of an overnight patient is calculated by subtracting the date the patient is admitted from the date of separation and deducting days the patient was on leave.

In 2015, a MAU was established in a large quaternary hospital in Southeast Queensland, Australia. The MAU was co-located in the ED, to improve emergency access and outcomes for medical patients presenting via the ED. The impetus for change followed a period of clinical redesign within the ED that achieved sustainable improvements in overall four-hour discharge compliance, but not for the medical patient cohort. Early senior clinician input is one of the factors considered critical to the success of the model as well increased nursing, medical and allied health staffing to deliver a model of care designed on six key principles:

1. Patient centred care;
2. Clear governance in terms of multidisciplinary team review;
3. Cooperation and co-location with ED (physically co-located or staff co-located);
4. Consistent clinical access (e.g. to “reliably decant MAU, providing consistent access to newly admitted patients”);
5. Continuity of care (e.g. minimisation of multiple handovers between medical teams for longer-staying patients); and.
6. Coordination of acute patient flow (Early Patient Intervention Centre Operational Brief, 2016, internal study site document)..

Geographic and resource constraints limited the bed capacity of the MAU to 8 beds in 2015 and re-location to another part of the hospital allowed an increase to 15 beds in 2016. Despite this increase, bed capacity was still less than the number of emergency medical admissions presenting to the ED (median of 18) on a daily basis (internal hospital data). Therefore, pragmatic decisions were made for a maximum 24-hour LOS in the MAU with strict application of clinical criteria (indications for referral to MAU) to maximise potential benefits of patient streaming to the MAU. The clinical criteria included indications and contraindications for referral to the MAU:

#### Indications

1. Clinical judgement of patient suitability for **rapid assessment and discharge** (e.g. uncomplicated chest pain, transient ischaemic attack) within 24 h.
2. Acutely unwell patients (i.e. **high acuity**) that would benefit from increased staffing and skill mix present in the MAU (e.g. upper gastrointestinal bleeding, pulmonary embolus)..

#### Contraindications

1. Patients likely to require longer than 24-hour length of stay.
2. Patients with relative medical stability (e.g. patients unlikely to experience clinical deterioration requiring acute nursing or medical interventions in the first 24 h of admission).
3. Patients at higher risk of delirium or behavioural disturbance (e.g. dementia, some mental health disorders such as eating disorders)..

### Data sources

As per usual process at the study site, administrative clinical data is submitted to the Health Round Table, a national collaborative of almost 150 hospitals and health services across Australia and New Zealand. The Health Round Table was established in 1995 to collect large datasets for the purposes of benchmarking, comparing performance, and sharing innovations and produces a series of automated but customised benchmarking reports in key clinical areas [19]. A report of clinically coded data submitted by the study hospital to the Health Round Table for February 2016 and June 2016 was used to identify the study cohort.

### Participants

#### Inclusion

All medical admissions regardless of length of hospital stay during February 2016 (a summer month in the Southern hemisphere) and June 2016 (a winter month in the Southern hemisphere) were included in the analysis. A summer and winter month were chosen to capture seasonal trends in patient presentations; there were no significant changes to the role or structure of the MAU between study periods.

#### Exclusion

Patients were excluded if they were transferred to the Emergency Short Stay Unit (SSU), Intensive Care Unit (ICU), or Coronary Care Unit (CCU) at the start of their admission as these patients are not suitable for the MAU. Patients were also excluded if they had an acute illness, chronic illness, or signs and symptoms of infection which required isolation, as these beds are allocated in a different manner. Finally, patients who discharged themselves against medical advice and those that were admitted to the hospital via interhospital transfer were also excluded.

### Data Linkage

Admissions were identified from the hospital information system and reporting datasets that are used to determine hospital funding (i.e. from the Health Round Table database described above). The patient hospital record number, age, gender, date and time of admission, length of stay, and discharge unit were extracted by a hospital administrator for all patient admissions from the ED in February 2016 and June 2016 for inclusion in the study. The hospital records department then filtered this list to patient admissions where the discharge unit was listed as a general medical unit or the acute medical assessment unit (i.e. the MAU). The medical record number was used to link cases identified from this filtered list with corresponding integrated electronic medical records (EMRs) to allow the auditors to extract clinical data from this secondary data source. All corresponding medical and administrative records stored in the integrated EMR and pathology databases were available for review by the auditors. Patient cases were de-identified in the data collection database.

### Audit Procedure

**Fig. 1:**
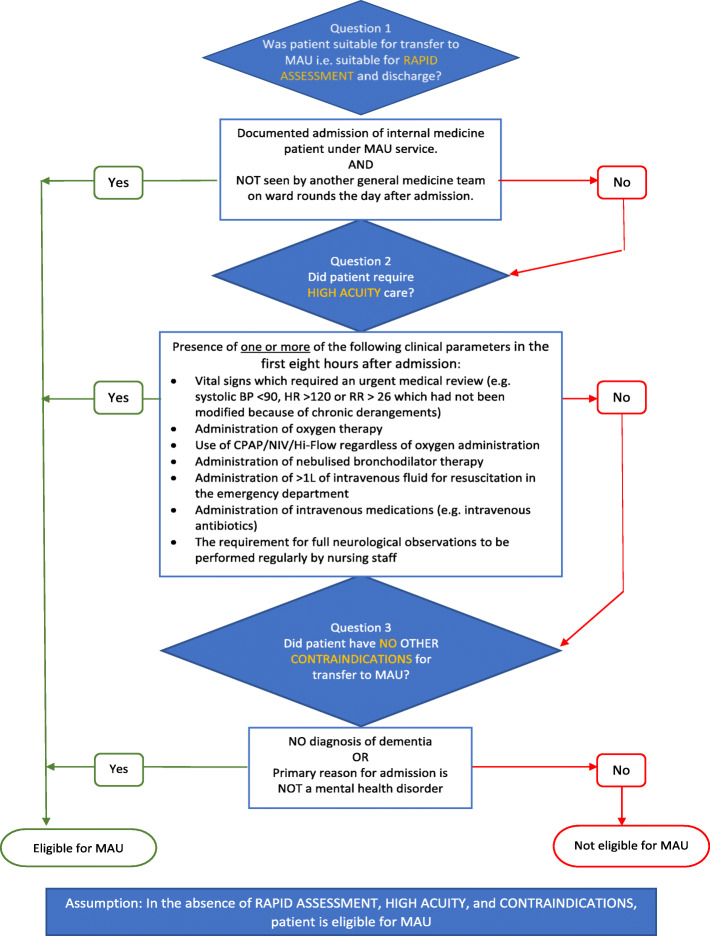
Audit Protocol

For the purpose of the medical record audit, clinical criteria rules were based on the MAU principles and defined through a consensus-driven approach to express the indications and contraindications to MAU admission in a way that would allow for consistency in data extraction across auditors. Three auditors were recruited from the pool of junior medical officers rotating through the MAU during the study period and trained by the Senior Medical Clinician on the research team in the process for extraction of relevant clinical data from the integrated EMR. To address potential bias, each auditor independently audited the same five cases against the predefined rules, with consistent results obtained – at which point the auditors progressed to review of cases for inclusion in the study. Auditors met collectively with the Senior Clinician on a regular basis during the process to discuss uncertainty and harmonise approaches to interpreting difficult cases. The dataset was divided equally between the three auditors who were then tasked with answering four key questions as outlined below. The audit decision-making flowchart is presented in Fig. 1.

### Data Collection

Data collected from the patient medical records included: age in years (admission date minus date of birth), gender as identified in record (male, female, other), diagnostic-related group (DRG) code and corresponding disease condition group at time of admission, admission date (day-month-year), and discharge date (day-month-year). In addition, data was collected on variables related to the MAU clinical criteria admission (indications and contraindications) as presented in the Audit Protocol (Fig. 1). Determination of eligibility for MAU was coded as 1 (yes) or 2 (no). Data was cleaned prior to analysis to remove duplicates, correct data entry errors, and standardise text descriptions.

### Outcome Measures

The primary outcome for this study was the proportion of admissions that adhered to the MAU admission criteria. Adherence with the MAU criteria were defined as either a patient who was transferred to MAU and met the High Acuity or Rapid Assessment INDICATION criteria (true positive), or met the CONTRAINDICATION criteria for the MAU ward and were not transferred to the MAU (true negative). Patients who had neither indication nor contraindication to MAU transfer were assumed to have a ‘PREFERENCE for MAU’.

A secondary outcome was admitted length of stay. This was defined as the time from admission to the general medical or MAU unit until discharge to investigate patient length of stay in relation to the different MAU principles.

### Statistical analysis

Descriptive statistics summarised total numbers of patients transferred to the MAU according to adherence with different MAU criteria (INDICATION, CONTRAINDICATION, PREFERENCE for MAU). For each criterion, exact binomial testing was applied to compare the observed percentage of patients transferred to the MAU with expected outcomes under random allocation (i.e. assuming a null hypothesis of 50 % transferred to the MAU). Overall adherence with MAU criteria was calculated as the sum of true positive and true negative patients, divided by the total number of admissions audited.

Follow up analysis looked at admissions that met the MAU indication criteria. Transfer outcomes were summarised separately for Acuity and Rapid Assessment groups. Admissions that satisfied both Acuity and Rapid Assessment criteria were assigned to the Acuity group. Logistic regression was then used to examine the influence of indication criteria and time of admission (Ordinary hours, After hours) on the likelihood of MAU transfer. These factors were included in the model as independent categorical factors and as a two-way interaction term. Additional independent variables were included to account for potential confounding from different auditors and season (Summer, Winter). Regression outcomes were reported as adjusted odds-ratios of MAU transfer. Differences in the secondary outcome were analysed using a two-proportion z-test. For all analyses, hypothesis testing was based on a 5 % level of statistical significance.

## Results

### Participant Demographics

**Fig. 2:**
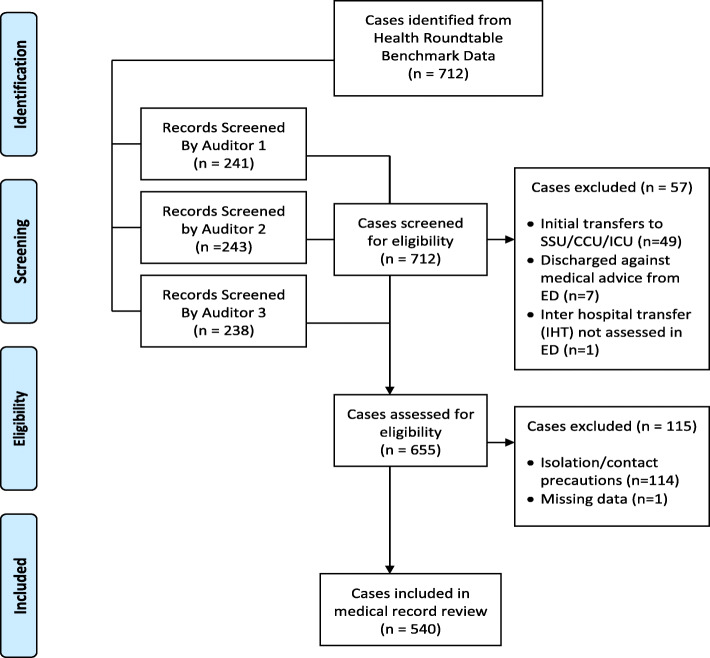
Participant Flow Chart. [adapted from Moher, Liberati, Tetzlaff, et al. 2009 [20]]

Of 712 patients identified from the filtered Health Round Table Benchmark Data and audited against the predefined inclusion and exclusion criteria, 540 cases were included in the medical review (Fig. 2). The cohort was nearly an equal split between males (*n* = 269) and females (*n* = 271) with a median cohort age of 67 years (range 16–99 years). There was much variety in the primary diagnoses across the cohort. The top five conditions, accounting for 65 % of the cohort, were disorders of the nervous system (including dementia-related cases) (22 %), cardiovascular and circulatory systems (17 %), respiratory system (11 %), digestive system (8 %), and musculoskeletal system (8 %).

### Primary Outcome

Of the total of 540 included patients, 386 (71 %) patients were deemed to meet the MAU eligibility admission criteria as presented in Table 2. Among patients with MAU indications, 66 % were correctly transferred (95 % CI: 61 to 71) to the MAU; this estimated sensitivity was statistically significant when compared with random allocation (*p*-value < 0.001). Transfer outcomes for patients with contraindications were subject to higher uncertainty, with a high proportion of these patients incorrectly transferred to MAU (73 % transferred; 95 % CI: 50 to 89 %; *p*-value = 0.052). Follow up analysis of these patients revealed that all were subsequently discharged from general medical wards. Excluding the PREFERENCE FOR MAU group, overall adherence with MAU criteria was 64 % (260/408; 95 % CI: 59 to 68 %). Of audited patients belonging to the PREFERENCE for MAU group, 61 % had a MAU transfer recorded (95 % CI: 52 to 69 %).

**Table 2:** Comparison of MAU transfer status (Yes/No) and MAU classification based on independent audits of ED admissions. CI: Confidence interval

MAU Transfer	MAU Classification (*n* = 540)		
	Indications for MAU	Contraindications for MAU	Preference for MAU
No	132	6^b^	52
Yes	254^a^	16	80
Total	386	22	132
% Transferred (95 % CI)	66 % (61–71 %)	73 % (50–89 %)	61 % (52–69 %)
*p*-value	< 0.001	0.052	0.018

^a^True positive; ^b^True negative

#### Indications for MAU

Within the Indications for MAU group, 90 % of patients (349/386) met the Acuity Indication criteria, of which 66 % (95 % CI: 61 to 71 %) were correctly transferred to the MAU (Table 3). Adherence with the MAU eligibility criteria were similar among Rapid Assessment patients; however smaller numbers in this group lead to greater estimate uncertainty (65 %; 95 %CI: 47 to 80 %).

**Table 3:** Sensitivity by MAU indication

MAU Transfer	Acuity	Rapid Assessment
No	119	13
Yes	230	24
TOTAL	349	37
% Transferred (95 % CI)	66 % (61–71 %)	65 % (47–80 %)
*p*-value	< 0.001	0.099

Logistic regression outcomes are summarised in Table 4. Results indicated that the likelihood of MAU transfer was similar between clinical indications (Adjusted odds ratio: 1.18; 95 % CI: 0.50 to 2.91; *p*-value = 0.711). In contrast, admissions during ordinary hours were more likely to be transferred to the MAU than admissions from the ED after hours (Adjusted odds ratio: 2.09; 95 % CI: 1.18 to 3.84; *p*-value = 0.014). Interaction between time of admission and clinical indication was not statistically significant (Adjusted odds ratio: 0.57; 95 % CI: 0.12–2.92; *p*-value = 0.484), suggesting that the likelihood of MAU transfer during ordinary hours was similar for Acuity and Rapid Assessment groups.

**Table 4:** Outcomes of logistic regression fitted to ED admissions that met clinical indications for MAU transfer (*n* = 386)

Variable	Number of admissions audited	Number transferred to the MAU (%)	Adjusted odds ratio Estimate (95 % CI)	*p*-value
Clinical indication				
Acuity	349	230 (66)	1.18 (0.50 to 2.91)	0.711
Rapid Assessment	37	24 (65)		
Time of admission				
After hours	295	185 (63)	2.09 (1.18 to 3.84)	0.014
Ordinary hours	91	69 (76)		
Season				
Summer	212	141 (67)	0.90 (0.58 to 1.38)	0.622
Winter	174	113 (65)		

Note: After Hours = (a) Evenings 1600–2200; (b) Weekends 0800–2200; (c) Night (7 days) 2200 − 0800

### Secondary Outcome

*Length of Stay*.

A larger proportion of patients discharged by the MAU team stayed in hospital for less than 24 h: 55.6 % for the MAU team compared with 10.5 % for other teams, giving an estimated percentage point difference of 45.1 % (95 % CI: 33.2 to 57.0 %) (Table 5). The percentage point difference was larger in patients who were not transferred to MAU (54.6 %, 95 % CI: 38.3 to 75.9 %, *p* < 0.001) than MAU transfer patients (37.7 %, 95 % CI: 22.6 to 52.9 %, *p* < 0.001).

**Table 5:** Proportion of patient discharged within 24 h, for patients discharged by the MAU team versus other medical teams

	Discharged by MAU team		Discharged by other teams	
MAU Transfer	n*	Discharged in ≤ 24 hours (%)	n	Discharged in ≤ 24 hours (%)
No (*n* = 190)	30	22 (73.3)	160	26 (16.3)
Yes (*n* = 350)	51	23 (45.1)	299	22 (7.4)
Total (*N* = 540)	81	45 (55.6)	459	48 (10.5)

*n is the total number of patients in each category

## Discussion

The purpose of this study was to determine the effectiveness of a consensus-derived set of clinical criteria for patient streaming from the ED to the MAU within the highly capacity-constrained environment of a large quaternary hospital. We found an overall adherence with MAU criteria of 64 %; however, an unexpected finding was that 73 % (n = 16/22) of patients with contraindications to MAU admission were inappropriately admitted to the MAU. These patients were subsequently transferred to other medical wards in the hospital, from which they were all discharged. The rate of inappropriate admissions to the MAU in our study significantly overwhelms findings from an earlier study of 100 consecutive medical admissions to an Emergency Short Stay (ESS) ward [21] which found that a little over a quarter of all admissions to ESS were found to be inappropriate. Hartley, et al. [21] concluded that the ESS was not always being used appropriately according to the admission criteria defined by the ward managers because although 68 % of patients were discharged home from ESS, 32 % of patients were later transferred to a different ward. Patient demographics in this study were similar to findings by Harley, et al. [21] in that most patients were admitted with respiratory or cardiac conditions.

When looking for explanatory factors we found that admissions during ordinary hours were more likely to be transferred to the MAU than admissions from the ED after hours. Possible reasons to account for this may be related to the discharge policies of the MAU, the higher staffing levels in the hospital, and/or differences in staff involved in bed allocation decisions between daytime hours and after hours. One of the pragmatic guidelines for admission to the MAU was a maximum 24-hour length of stay, which necessitated rapid assessments and diagnostic tests to appropriately treat patients and either transfer them out of the MAU or discharge them with instructions to return to the outpatient clinic for follow up. This intense clinical activity was more likely to be supported during ordinary hours because of higher availability of staff such as pathology, radiology, pharmacy and allied health. In addition to the higher levels of multidisciplinary clinicians to assist in rapid assessment and diagnosis during the day, the staffing profile during ordinary hours is different than after hours. For instance, during ordinary hours there are a team of bed managers constantly assessing bed capacity, organising patient transfers into and out of units, and were more available to consult with nurse managers and medical clinicians regarding appropriate bed allocation. After hours, there is usually a sole decision maker with respect to bed allocation, the After-Hours Nurse Manager. After hours, bed allocation decisions may more likely be based on pragmatic reasons such as bed capacity and availability. This rationale confirms earlier studies on bed management. In a seminal study of bed management practices based mainly on qualitative interviews with 40 staff (bed managers, senior medical clinicians and nurses) and shadowed observations of three bed managers, Green and Armstrong [22] concluded that bed shortages influenced clinical decisions more than clinical decisions influenced bed shortages.

In exploring other explanatory factors, previous research suggested that seasonal variation could account for ED overcrowding and patient hospital admissions [4–6]. Our findings showed no significant differences in MAU admissions between the winter and summer data collection periods; however, we did find an unexpectedly high proportion of these patients inappropriately transferred to MAU. This might be an unintended consequence of using MAUs as an overflow catchment for patients who would not normally meet the MAU admission criteria.

This raises vital questions about other possible influences in addition to clinical criteria rules, outlined in the MAU admission operational brief, in directing patient flow to the MAU in order to account for the lack of consistent adherence to the MAU admissions criteria. In other words, other factors may be driving the decision-making process. Decision-making by senior clinicians in general medicine is thought to be guided by clinical gestalt, a theory which posits that “healthcare practitioners actively organise clinical perceptions into coherent construct wholes” (p.6) [23]. This means that senior clinicians can tacitly make clinical decisions without having complete information and can construct generalisable solutions that can transfer from one problem encountered to another [23]. Thus, with experience, there is less reliability on strictly enforced clinical criteria rules or evidence-based guidelines when making decisions. This is supported by [24] who explored 366 discharge decisions made by almost 90 % of the experienced ED physicians in a large, high acuity, metropolitan hospital in Canada with 68,000 admissions per year. A real-time survey using qualitative interviews revealed that ED physicians reported using clinical judgement more than evidence when making decisions to discharge or admit patients (87.6 % vs. 12.4 %, respectively) [24]. While collecting data on patient outcomes was beyond the scope of our study, we note that [24] found no adverse events in patients decisions based on evidence or clinical judgement.

While the decision to admit and treat individual patients is traditionally the responsibility of doctors, it is nurses who are largely in charge of managing overall bed capacity and patient flow. In an ethnographic study involving non-participant observations and in-situ interviews of 40 nurses working in a large university hospital in the United Kingdom, Allen [25] described a process of ‘match-making’, where bed managers and nurses negotiated the needs of patients and essentially re-shaped the organisation to create bed accommodation where necessary to optimise hospital bed capacity. While, clinical judgment is generally established as the process in making decisions to transfer patients to MAU, our findings suggest that we cannot assume that clinical gestalt works in isolation. Contextual factors may play a role. Bed availability, time of day, staffing levels, and discipline profile of decision-making staff rostered during ordinary hours and after hours also account for the degree of effectiveness of directing patient flow. Alongside these operational contextual factors, another potential explanation is a lack of intra- and inter-disciplinary agreement or transparency on whether patients meet the indications or contraindications for the MAU. Clinical assessment and referral involves cognitive processes such as negotiation, clinical reasoning, and decision-making [26]. Further, nurses and medical officers not only use but also value different types of knowledge and adopt different roles in clinical decision-making [27]. This questions whether decisions to transfer are based on specific clinical criteria, a more global clinical gestalt, some other cognitive processes underpinning decision-making, or a unique combination of them all. What is missing in the equation is *how* the decisions are made and *by who* and what are the drivers or determinants of decision making. We can only answer these questions by further exploring the clinicians’ decision-making process. Also, for consideration is establishing whether administrators are willing to accept that a certain level of inefficiency is inherent in the process of streaming patients from the ED to the MAU in the complex environment of a large and busy hospital system, or whether improving the effective utilization of a MAU is more desirable. We agree with Suthers and colleagues [1] that those considering the establishment of a MAU need to consider other factors that may mitigate the improvements in minimising roadblocks to discharge, the organisation of allied health staff, and the number of transfers of care. This study supports the early recommendation by Cooke and colleagues [28] in that a detailed understanding, as opposed to simple solutions, are required to address the complex hazards and issues surrounding the transfer of care between the ED and medical areas, including to MAUs.

### Limitations

One of the limitations of retrospective medical chart review audits is the completeness of the data or missing data. We were able to retrieve medical records for all patient cases that met the eligibility criteria at the start of the study. Only one record was found to have key missing data. In this situation, the patient case was excluded from the study. Panachek [29] recommends that if less than 5 % of charts are missing, this can be ignored as a source of bias.

We did not collect data on health status changes while patients were awaiting transfer from the emergency department, on patient clinical outcomes at discharge, or on the organisational context of the hospital setting. This information may have provided further insights on causal mechanisms embedded within the particular context of the hospital and the social processes underpinning clinician decision making to transfer patients to the MAU, such as described in realist evaluation [30]. However, as our key concern for this first phase of our two phased-study was the effectiveness of the clinical criteria in decisions to transfer appropriate patients to the MAU, collecting extraneous patient health data was beyond the scope of this study.

While collecting data from a single site may have implications for generalisability, using Australian Institute of Health and Welfare (AIHW) data [18], this study was able to demonstrate the comparability of the study site to other principal referral public hospitals in emergency department presentations, number of medical patient hospital admissions and diversity of medical patient cohort, making the findings applicable to similar settings.

Finally, there are acknowledged challenges with conducting secondary analyses of existing data. The obvious benefits are that it is less time-consuming and less expensive than actually collecting the data. Disadvantages are that the data was not originally collected to address the current research question and some data variables that could be useful for the current study may not have been collected [31] or the original data source may be based on incomplete or inadequate data reporting [16]. For this study, data was obtained from the Health Round Table dataset which was collected in accordance with guidelines detailed by the Health Round Table to ensure that data collected from all participating hospitals was collected in a consistent way so that benchmarking was possible [19].

## Conclusions

There is little published data on the effectiveness of clinical criteria in ensuring the ‘right’ patients reach the MAU. We show that while clinical criteria and clinical judgement are the established approach to patient streaming, other contextual factors override clinically based decisions, reducing the efficiency in the use of MAUs as intended. Despite the less than appropriate patients that might be admitted to the MAU, these patients are still discharged from the MAU in less than 24 h, with patient disposition being discharge from hospital or transfer to another hospital ward. Our study was able to establish the sensitivity and specificity of criteria used in decision making to stream patients from the ED to the MAU and can assist health service planning aimed at improving efficiency in patient flow and patient management for medical patients presenting to the ED. More research exploring other explanatory variables influencing efficiency of patient streaming to MAUs and determinants of clinician decision making are needed. As decision making is a human process, looking at human factors as well as system factors may provide more insight on how decisions are made and who makes them in order to inform organisations how best to allocate resources (people, space, things) to make an MAU more efficient in the larger scheme of patient flow.

## Data Availability

The de-identified datasets generated and/or analysed during the study may be available if appropriate permissions are obtained (by those seeking to access the data) from the data custodians with appropriate ethical and governance approval. The Senior Clinician co-investigator (JdL) can be contacted at Julian.deLooze@health.qld.gov.au for further information regarding access to the dataset.

## Abbreviations

ED: Emergency Department
EMR: Electronic Medical Record
LOS: Length of Stay
MAU: Medical Assessment Unit

## Declarations

### Authors’ Information

Sonya R Osborne RN, PhD, MN (Research), GradCert (High Educ), GradCert Nursing (Perioperative), BS Psych, BSN, FACORN.

Associate Professor (Nursing), School of Nursing and Midwifery, Centre for Health Research, Institute for Resilient Regions, University of Southern Queensland, Ipswich, Queensland, Australia 4305.

Adjunct Associate Professor, Implementation Science, Australian Centre for Health Services Innovation, School of Public Health and Social Work, Queensland University of Technology, Kelvin Grove, Queensland 4059 Australia.

Helen Cleak PhD, MSW, BA, DipSocStuds.

Adjunct Associate Professor, Department of Community and Clinical Health, La Trobe University, Melbourne, Victoria, Australia 3086.

Nicole White PhD (Statistics), BMath, BAppSci (Hons).

Research Fellow, Australian Centre for Health Services Innovation, School of Public Health and Social Work, Queensland University of Technology, Kelvin Grove, Queensland 4059 Australia.

Xing Lee PhD, BMath, BAppSci (Hons).

Visiting Fellow, Australian Centre for Health Services Innovation, School of Public Health and Social Work, Queensland University of Technology, Kelvin Grove, Queensland 4059 Australia.

Anthony Deacon MBBS, BSc, GCELead, GCAcadPrac, MBus.

Medical Registrar, Department of Internal Medicine and Aged Care, Royal Brisbane and Women’s Hospital, Metro North Hospital and Health Service, Herston, Queensland 4029 Australia.

Associate Lecturer, School of Medicine, The University of Queensland, St. Lucia, Queensland, 4067 Australia.

Adjunct Fellow, School of Electrical Engineering and Computer Science, Queensland University of Technology, Kelvin Grove, Queensland, 4059 Australia.

Julian W M de Looze MPH, MBBS, BSc(Hons), FRACP.

Staff Specialist, Department of Internal Medicine and Aged Care, Royal Brisbane and Women’s Hospital, Metro North Hospital and Health Service, Herston, Queensland 4029 Australia.

Senior Lecturer, School of Medicine, The University of Queensland, St. Lucia, Queensland, 4067 Australia.
